# Fabrication of Nerve Growth Factor Encapsulated Aligned Poly(ε-Caprolactone) Nanofibers and Their Assessment as a Potential Neural Tissue Engineering Scaffold

**DOI:** 10.3390/polym8020054

**Published:** 2016-02-19

**Authors:** Jue Hu, Lingling Tian, Molamma P. Prabhakaran, Xin Ding, Seeram Ramakrishna

**Affiliations:** 1 College of Textiles, Donghua University, Shanghai 201620, China; a0120192@nus.edu.sg; 2 Center for Nanofibers and Nanotechnology, Department of Mechanical Engineering, Faculty of Engineering, National University of Singapore, 2 Engineering Drive 3, Singapore 117576, Singapore; mpetian@nus.edu.sg (L.T.); nanotechmpp@gmail.com (M.P.P.); 3 Guangdong-Hongkong-Macau Institute of CNS Regeneration (GHMICR), Jinan University, Guangzhou 510632, China

**Keywords:** emulsion electrospinning, neural tissue engineering, neural extension, aligned fiber, sustained release

## Abstract

Peripheral nerve injury is a serious clinical problem to be solved. There has been no breakthrough so far and neural tissue engineering offers a promising approach to promote the regeneration of peripheral neural injuries. In this study, emulsion electrospinning technique was introduced as a flexible and promising technique for the fabrication of random (R) and aligned (A) Poly(ε-caprolactone) (PCL)-Nerve Growth Factor (NGF)&Bovine Serum Albumin (BSA) nanofibrous scaffolds [(R/A)-PCL-NGF&BSA], where NGF and BSA were encapsulated in the core while PCL form the shell. Random and aligned pure PCL, PCL-BSA, and PCL-NGF nanofibers were also produced for comparison. The scaffolds were characterized by Field Emission Scanning Electron Microscopy (FESEM) and water contact angle test. Release study showed that, with the addition of stabilizer BSA, a sustained release of NGF from emulsion electrospun PCL nanofibers was observed over 28 days. [3-(4,5-dimethylthiazol-2-yl)-5-(3-carboxymethoxyphenyl)-2-(4-sulfophenyl)-2*H*-tetrazolium, inner salt; MTS] assay revealed that (R/A)-PCL-NGF and (R/A)-PCL-NGF&BSA scaffolds favored cell growth and showed no cytotoxicity to PC12 cells. Laser scanning confocal microscope images exhibited that the A-PCL-NGF&BSA scaffold increased the length of neurites and directed neurites extension along the fiber axis, indicating that the A-PCL-NGF&BSA scaffold has a potential for guiding nerve tissue growth and promoting nerve regeneration.

## 1. Introduction

The incidence of peripheral nerve injury has a significant upward trend with the increasing of the mechanization degree in industrial production and the development of transportation. Nerve defects caused by nerve injury are a difficult problem in clinical treatment. For small nerve defects, spontaneous recovery of a nerve can occur, while it remains a major challenge for surgeons to repair large nerve defects (>10 mm) [1]. Nerve autograft is the best way of repair large peripheral nerve defect. However, due to the limited resource of autografts and the inevitable sensory loss of the donor area after donor nerve graft, it is difficult to meet the increasing requirement for peripheral nerve defect repair. To overcome the shortages of autologous nerve grafts, the development of biomaterial scaffolds for nerve regeneration and the recovery of neural function are imperative. Many researchers have tried to develop more effective biomaterials to create an alternative to the autograft and promote nerve regeneration [2].

Electrospinning is an extremely simple and powerful approach to fabricate polymeric fibers in the sub-micrometer scale, ranging from about 50 nm to several microns. Meanwhile, the native extracellular matrix (ECM) is a three-dimensional scaffold consisting of polysaccharide and protein fibers (collagen and elastin fibers, *etc*.), which is also in the nanoscale from tens of nanometers to hundreds of nanometers [3]. Therefore, electrospun nanofibers can mimic ECM structure, providing a three-dimensional space and more adhesion sites for the growth of cells and can be used as the tissue engineering scaffold [4]. What’s more, electrospun polymeric drug loaded nanofibers are capable of serving as both drug delivery vehicles and tissue engineered scaffolds, as the drug release properties can be finely tailored through modulating the morphology, porosity, and composition of the drug incorporated nanofibers [5,6]. At the same time, the large surface areas and interconnected pores of the nanofibrous mats are favorable for cell/tissue ingrowth [7,8].

It is known that surface topography of the scaffolds plays a critical role in regulating cell behaviors, such as adhesion, migration, and differentiation, both *in vitro* and *in vivo* [7]. Literatures have proposed that the aligned nanofibrous scaffolds were able to instruct the cultured cells directionally growth, resulting in the elongation and alignment of cells along the major axis of the nanofibers [8,9,10]. Particularly, aligned fibrous scaffolds with topographical cues have shown a superior capacity in directing neurite outgrowth and extension, and successfully connecting target injured peripheral nerve tissues [8,9].

Neurotrophic factor, such as Nerve growth factor (NGF), has beneficial effects on neuronal cell survival and function [11]. Thus, it is widely used for nerve regeneration. However, NGF is chemically unstable and has short half-life *in vivo*. In addition, the dosage of growth factors required to obtain biological activity was quite small, which is usually in the picograms to low nanogram range [12], and excess usage of NGF might cause undesirable side effects [13]. In order to administer NGF in a stable low concentration for the recovery of nerve injury, it is necessary to find a carrier for the encapsulation of NGF, which can not only prevent the growth factor from rapidly eliminating, but also promote tissue regeneration in the corresponding period with the controlled release of the suitable dose of NGF [7,14]. Nanofibers with core-shell structure can meet the above requirements, where the protein forms the core, while the polymer becomes shell, thus protecting the bioactivity of protein and lengthening the release time of it. In recent decades, coaxial electrospinning was reported to be a useful technique in fabricating core-shell nanofibers, in which protein was encapsulated as the core [15]. Tian *et al.* [7] incorporated NGF along with silk fibroin into poly(lactic acid) nanofibers successfully. During the coaxial electrospinning process, a complex dual-capillary spinneret apparatus is required. Alternatively, emulsion electrospinning has been developed to prepare core-shell structured nanofibers as the drug delivery vehicles [16]. Recently, emulsion electrospinning technique was applied successfully for the encapsulation of drugs, proteins, and growth factors into the inner core of the nanofibers [16,17,18]. Although different drugs/proteins have been encapsulated within various polymers by emulsion electrospinning technique, there is a dearth of studies on the influence of surface topography of drug loaded emulsion electrospun nanofibers on their release properties.

Polycaprolactone (PCL) is a kind of biodegradable polyester certified by the U.S. Food and Drug Administration (FDA). Electrospinning of PCL and its blends have been widely used for tissue engineering, due to its good biodegradability, biocompatibility, and minimal inflammatory reactions [19]. We previously demonstrated that optimized PCL nanofiber morphologies with alleviated burst release and sustained drug release can be achieved by emulsion electrospinning [18]. According to our previous research, PCL was a superior biomaterial for generating drug loaded tissue engineering scaffolds [18]. Zhu *et al.* [8] found out that biodegradable PCL scaffolds were suitable for the survival and differentiation of PC12 neural like cells with a low risk of being rejected by human body. Therefore, PCL was chosen as the matrix material in this study.

Traditional electrospun fibers usually arranged randomly, but the distribution of nerve cells, vascular endothelial cells, and muscle cells in the human body has certain direction. Therefore, it has very important significance for inducing cultured cells to grow along a certain direction. Recently, it has become one of the research hot spots in the field of tissue engineering, where aligned fibers are prepared through electrospinning method to provide contact guidance to seeded cells on the scaffolds. In the present study, we fabricated novel BSA, NGF, and NGF&BSA incorporated PCL core-shell random and aligned nanofibrous scaffolds by emulsion electrospinning. The release behavior of NGF from emulsion electrospun (R/A)-PCL-NGF&BSA nanofibers showed a low burst release and followed by 28 days sustained release of NGF, matching a typical nerve regeneration period of around four weeks [12,20,21]. The bioactivity of NGF released from nanofibers was confirmed by rat pheochromocytoma (PC12) cells, which can respond specifically to NGF in the neuronal differentiation, including neurite outgrowth and expression of neuronal markers. We investigated the synergistic effect of topographic cue and the NGF-trigger on the neurite outgrowth of PC12 cells and explored the potential application of the (R/A)-PCL-NGF&BSA nanofibrous scaffolds as superior carriers in neural tissue engineering.

## 2. Materials and Methods

### 2.1. Materials

Poly(ε-caprolactone) (*M*_w_ 80 kDa), glutaraldehyde, sorbitan monooleate (Span 80), hexamethyldisilazane (HMDS), sodium dodecyl sulfate (SDS), Dulbecco’s modified eagle’s medium (DMEM/F12) were purchased from Sigma, Singapore. Chloroform (CHCl_3_) was purchased from Fisher Scientific Company, Loughborough, UK. Micro BCA Protein Assay Kit was purchased from Thermo Scientific (Rockford, lL, USA). Sodium hydroxide (NaOH) was bought from Merck (Singapore). Nerve growth factor (NGF 7S) and ChemiKine Nerve Growth Factor Sandwich ELISA kit were purchased from Millipore, Singapore. Rat pheochromocytoma (PC12) cells in the adherent type [PC12 Adh (ATCC^®^ CRL1721.1™)] were obtained from ATCC, Manassas, VA, USA, while fetal bovine serum (FBS), horse serum (HS), and trypsin/EDTA were purchased from Invitrogen/Gibco, Grand Island, NY, USA. [3-(4,5-Dimethylthiazol-2-yl)-5-(3-carboxymethoxyphenyl)-2-(4-sulfophenyl)-2*H*-tetrazolium, inner salt; MTS] was purchased from Promega, Madison, WI, USA. Alexa Fluor 594 Goat anti-Mouse IgG antibody was obtained from Invitrogen, Eugene, OR, USA.

### 2.2. Scaffolds Fabrication

**Figure 1:**
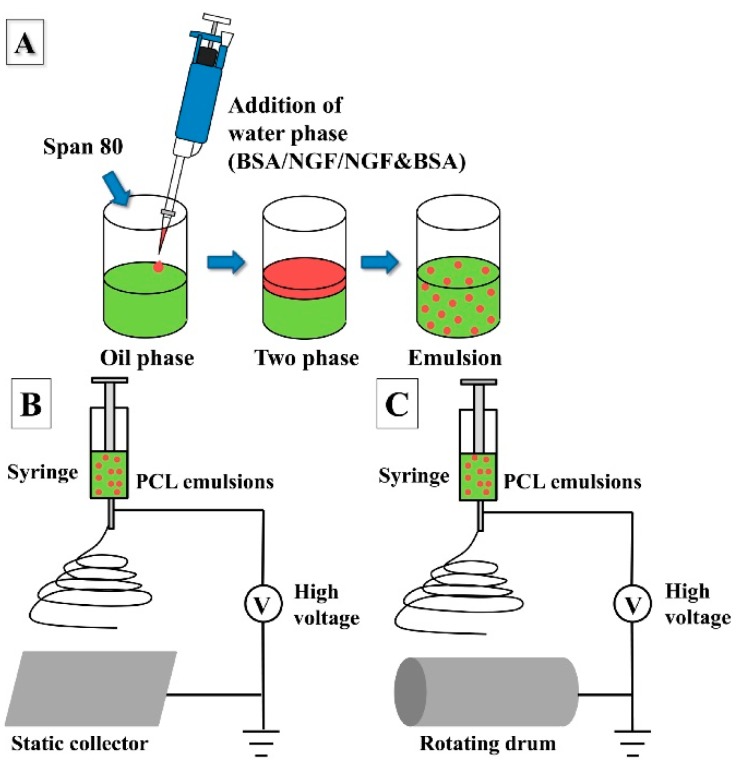
Schematic illustration of (**A**) emulsion preparation process, and the electrospinning set-up to produce (**B**) random and (**C**) aligned nanofibers. Red color represents water phase and green color indicates oil phase.

Random (R) and aligned (A) drug-loaded PCL nanofibers were fabricated via our lab’s electrospinning set-up as shown in Figure 1B,C respectively. In detail, the emulsion electrospinning method involved the use of two phases (Figure 1A): (1) an inner water phase (W) containing the solute to be incorporated, and (2) an outer organic phase (O) consisting of PCL/CHCl_3_ solution together with an emulsifying agent (Span 80). Table 1 listed different components for the preparation of various nanofibers. In brief, 8 μg NGF, 8 mg BSA, or 8 μg NGF together with 8 mg BSA were dissolved in 250 μL distilled water (DW) and then added dropwise to the above mentioned PCL/CHCl_3_ solution. Literature shows that the ratio of NGF:BSA ranges from 1/100 [22] to 1/10,000 [23], and in this study, we chose a ratio of 1:1000 (NGF:BSA) for the release and bioactivity study of the random and aligned NGF&BSA loaded nanofibers [(R/A)-PCL-NGF&BSA]. For the preparation of random PCL nanofibers, 400 mg PCL with appropriate amount of surfactant Span 80 were dissolved in 5 mL chloroform to form the 8% (*w*/*v*) PCL/CHCl_3_ solution. While for the fabrication of aligned nanofibers, the concentration of PCL was slightly increased to 9% (*w*/*v*) to make sure that random and aligned PCL nanofibers have roughly the same fiber diameter. Random and aligned pure PCL nanofibers [(R/A)-PCL], NGF or BSA incorporated emulsion electrospun PCL nanofibers [(R/A)-PCL-NGF, (R/A)-PCL-BSA] were also made under similar electrospinning conditions. All emulsions were stirred for about 2 h to allow the water phase evenly dispersed throughout the PCL/CHCl_3_ solution and then used immediately for electrospinning. Plain PCL nanofibers were also made by conventional electrospinning of 8% or 9% PCL in appropriate amount of CHCl_3_/Methanol (80:20) solutions as control. The syringe loaded with the solutions was fitted with 18 G blunt stainless needle and pumped at a flow rate of 1 mL/h by a syringe pump (KDS 100, KD Scientific, Holliston, MA) under a high voltage of 16 kV (Gamma High Voltage, Ormond Beach, FL, USA). Electrospun nanofibers were collected at a distance of 10 cm from the needle tip to a static collector for random nanofibers and to a rotating drum for aligned nanofibers.

### 2.3. Characterization of Scaffolds

#### 2.3.1. Observation of Field Emission Scanning Electron Microscope (FESEM)

Surface topography of emulsion electrospun nanofibrous scaffolds was examined via FESEM (Model S-4300, Hitachi, Tokyo, Japan) after sputter-coated with gold. The average diameter of the nanofibers from the SEM images was calculated with image analysis software (Image J, National Institutes of Health, Bethesda, MD, USA).

#### 2.3.2. Observation of Transmission Electron Microscopy (TEM)

The core-shell structure of the random and aligned PCL-BSA, PCL-NGF, PCL-NGF&BSA nanofibers was verified using transmission electronic microscopy (JEM-3010, JEOL, Tokyo, Japan) at 200 kV. To prepare a sample for TEM observation, a few nanofibers were collected on a carbon film-coated copper grid. For comparison study, the TEM images of plain PCL nanofibers were also observed.

#### 2.3.3. Measurement of Water Contact Angle

The water contact angles of nanofibrous mats were measured by VCA Optima Surface Analysis System (AST products, Billerica, MA, USA) to identify the effect of Span 80 existence on the hydrophilicity of electrospun nanofibrous mats. The nanofibers were collected on coverslips and placed on a testing plate. Subsequently, 0.05 mL of distilled water was carefully dropped onto the specimens. The images of water contact angles were recorded by a dynamic camera at 12 s after the droplets touched the surface of the nanofibrous mats. Three different points of each sample were measured carefully at ambient temperature.

### 2.4. Encapsulation Efficiency and In vitro Protein Release Study

Ten milligrams of BSA-loaded emulsion electrospun random and aligned PCL nanofibers [(R/A)-PCL-BSA] were immersed into 6 mL of 0.05 M NaOH solution (containing 0.5% *w*/*v* SDS) overnight at room temperature to achieve BSA extract solution. BSA standard curve at concentrations from 0 to 500 μg/mL were prepared according to BCA kit protocol. NaOH&SDS solution with the fiber were then centrifuged at 5000 rpm for 10 min. The supernatant (150 µL) was aspirated out in triplicate for BSA concentration determination using Micro BCA protein assay kit. In detail, 150 µL of the working reagent were mixed with the supernatant sample, placed in a 96-well plate and incubated for 2 h at 37 °C. Subsequently, the absorbance was read at 562 nm using a microplate reader (Varioskan Flash Multimode Reader, Thermo Scientific). The encapsulation efficiency (EE%) was calculated according to the following equation:

EE% = [(amount of detected BSA)/(amount of BSA theoretically incorporated)] × 100(1)

Because the NGF theoretically incorporated amount was very small (1.6 µg/mL in the electrospinning solution), which is only 1/1000 of the BSA added (1.6 mg/mL in emulsions). We could not extract and detect NGF from nanofibers by the conventional method, therefore EE of BSA was used as an indicator to estimate the EE of NGF according to the reference published by Zhu *et al.* [24].

To investigate the release profiles of BSA from emulsion electrospun R-PCL-BSA and A-PCL-BSA nanofibers, 10 mg of BSA loaded nanofibrous mats were incubated in 1 mL of phosphate buffered saline (PBS, pH 7.4) supplemented with 0.05% Tween 20, a common protein stabilizer [25,26,27], which was placed in a microcentrifuge tube and the tubes were put onto a thermostatic shaker at 37 °C. At predetermined time intervals, the PBS was completely removed from each sample for analysis and 1 mL of fresh PBS was refill for continuous incubation. The amount of BSA released into PBS buffer was determined by Micro-BCA protein assay kit and tested by a microplate reader (Varioskan Flash Multimode Reader, Thermo Scientific) at a wavelength of 562 nm, and the release results of BSA were expressed by cumulative release as a function of the release time:

Cumulative amount of release (%) = *M_t_/M_a_* × 100 (2)

where *M_t_* is the amount of BSA released at time t and *M_a_* is the actual amount of drug incorporated into nanofibers. The samples at all time points were run in triplicate.

For NGF release studies, 10 mg of NGF loaded nanofibrous mats, including R-PCL-NGF, A-PCL-NGF, R-PCL-NGF&BSA, and A-PCL-NGF&BSA were incubated under the same conditions as BSA release. At regular intervals, the release medium was completely taken out and exchanged with fresh buffer for continuous incubation. Release solution containing NGF was collected and kept at −20 °C until analysis by enzyme-linked immunosorbent assay (ELISA). The antibody in the NGF Sandwich ELISA kit specifically reacts with the β-subunit of NGF in the 7S form. NGF release experiment at every time point was performed in triplicate.

### 2.5. Cell Culture and In vitro Cytotoxicity Study

Dulbecco’s modified eagle’s medium (DMEM/F12; Sigma) supplemented with 15% horse serum (HS; Gibco), 2.5% fetal bovine serum (FBS; Gibco), and 1% antibiotic/antimycotic solution (Sigma) was used as the normal Growth Medium. PC12 cells (CRL-1721.1, from ATCC) were cultured in a 75 cm^2^ cell culture flask with Growth Medium and incubated at 37 °C in an incubator containing 5% CO_2_ until sufficient confluence. The Growth Medium in flask was changed once in every two days. All the nanofibrous samples were sterilized under UV light and placed in the 24-well plates pressed with a stainless steel ring to ensure complete contact of the nanofibrous scaffolds with wells, then washed thrice with PBS, and subsequently immersed in medium until cell seeded. After cells cultured for three days, PC12 cells were detached from the flask using 1× trypsin/EDTA, counted under optical microscope using hemocytometer and seeded on coverslips (CS), (R/A) PCL, (R/A)-PCL-BSA, (R/A)-PCL-NGF and (R/A)-PCL-NGF&BSA nanofibrous scaffolds at a density of 1.0 × 10^4^ cells/well, and then cultured in 1 mL of Growth Medium in the 24-well plates.

MTS assay was used to measure the cytotoxicity after PC12 cells seeded on CS and nanofibrous scaffolds for two, four, six, and eight days of culture. Briefly, the cell-nanofibrous scaffolds were rinsed with PBS to remove unattached cells and incubated in serum free medium with 20% MTS reagent for 3 h at 37 °C. The reaction medium of every sample was aliquoted into 96-well plates and the absorbance of each well was measured at 490 nm wavelength using a spectrophotometric microplate reader (Varioskan Flash, Thermo Scientific).

### 2.6. Differentiation Study of PC12 Cells

PC12 cell were used to assess the bioactivity of NGF release from electrospun NGF-loaded PCL nanofibers since they can differentiate toward a neuronal phenotype under stimulation of NGF [1,7,10]. For the observation of PC12 cell differentiation, cells were cultured in a NGF(+)Differentiation Medium composed of DMEM/F12, 1% HS, 0.5% FBS, and 1% antibiotic/antimycotic solution with the addition of NGF at a concentration of 50 ng/mL. For comparison, cells were also cultured using the same above medium without NGF, which is referred as NGF(−)Differentiation Medium. Briefly, PC12 cells were seeded at a density of around 1.0 × 10^4^ cells/well on CS, (R/A)-PCL, (R/A)PCL-BSA, (R/A)PCL-NGF, and (R/A)PCL-NGF&BSA in a 24-well plate respectively and cultured in Growth Medium. After cell seeded for one day, the Growth medium in every well was replaced with NGF(+) Differentiation Medium or NGF(−)Differentiation Medium. 1 mL NGF(+)Differentiation Medium was added into the wells with CS, (R/A)-PCL and (R/A)PCL-BSA, while 1 mL NGF(−)Differentiation Medium was added into the wells with CS, (R/A)PCL-NGF, and (R/A)PCL-NGF&BSA. PC12 cells with one or more neurites equal to or longer than their soma length were considered as differentiated cells with positive response to bioactive NGF. The morphology of the differentiated PC12 cells was observed by both SEM and fluorescent microscopy.

#### 2.6.1. Morphology Study of Differentiated PC12 Cells by SEM

SEM was used to study the morphological changes of *in vitro* differentiated PC12 cells on different scaffolds after eight days of cell culture. The scaffolds with cells were rinsed thrice with phosphate buffer saline (PBS) and fixed with 3% glutaraldehyde for 3 h, followed by rinsing three times with distilled water and dehydration with increasing concentration of ethanol (50%, 70%, 90%, and 100%) for 15 min each. Finally, all scaffolds with cells were treated with HDMS and kept in room temperature overnight for air-drying. All the cells-scaffolds samples were observed by FESEM after sputter coating with gold.

#### 2.6.2. Immunofluorescence Staining

After eight days of cells cultured in NGF(+) or NGF(−) Differentiation Medium on different scaffolds, the immunostaining of cytoskeletal proteins α-Tubulin was carried out to observe the cell phenotype and neurite extension of differentiated PC12 cells. The reason for choosing α-Tubulin as the staining marker was based on former studies [28,29]. All the scaffolds were rinsed with PBS, fixed in neutral buffer formalin solution (Sigma) for 20 min and permeabilized with 0.25% Triton X-100. The nonspecific binding was blocked by incubating with 3% BSA in PBS for 90 min. Subsequently the samples were incubated with a neuronal-specific Mouse anti-α-Tubulin antibody (Invitrogen, Camarillo, CA, USA), followed by the staining of Alexa Fluor 594 Goat Anti-Mouse IgG (H + L) antibody (Invitrogen), and the nuclei was stained with DAPI (Thermo Scientific). The immunostained samples were mounted on glass slides and fluorescent images were taken using a laser scanning confocal microscope (Zeiss LSM700). Some samples incubated with PBS instead of anti-α-Tubulin antibody were used as the blank control group.

### 2.7. Statistical Analysis

All of the data were expressed as means ± standard deviation. Statistical analysis was carried out using one-way ANOVA (SPSS, Version 20) and a value of *p* < 0.05 was considered statistically significant.

## 3. Results and Discussion

### 3.1. Formation of Emulsion Electrospun Nanofibrous Scaffolds

For the emulsion electrospinning of random nanofibers, 8% (*w*/*v*) PCL was added to CHCl_3_ as the oil phase, while 9% (*w*/*v*) PCL was used for the preparation of aligned nanofibers. It is necessary to increase the concentration of PCL in aligned fiber preparation, since the high rotational speed of the drum results in thinner fibers. In order to compare the influence of scaffolds construction on neuron-like PC12 cells behaviors, other factors, such as the big difference in fiber diameter between random and aligned nanofibers should be avoided. Therefore, in this study, a higher concentration of PCL was used to produce the aligned nanofibers (Table 1).

### 3.2. Characterization of Nanofibrous Scaffolds

#### 3.2.1. Morphology Analysis of Nanofibrous Mats

**Figure 2:**
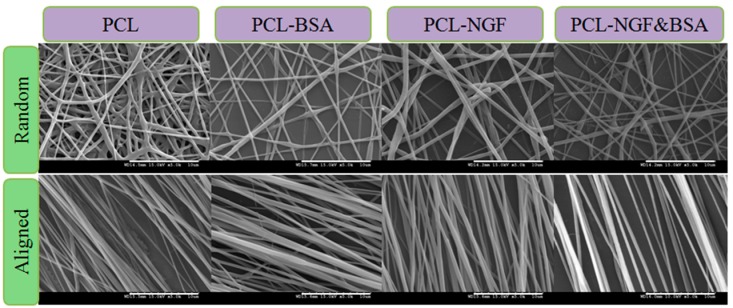
FESEM images of random and aligned PCL nanofibers, scale bar = 10 μm.

Figure 2 displays FESEM images of random and aligned PCL nanofibrous scaffolds which were collected on static foil and rotating cylindrical mandrel, respectively. The diameter distribution of different nanofibers is shown in Table 1. Obviously, nanofibers collected on rotating drum were approximately parallel to the longitudinal direction of the fiber and presented roughly the similar mean diameter as the random nanofibers. In addition, the standard deviation of the diameter of aligned fibers was smaller, indicating more uniform nanofibers were made in aligned structure. Similarly, several researchers have found out the fiber diameter and size distribution, as well as the orientation of fibers, can be modulated by the rotational speed and polymer concentration, and increasing the rotating rates resulted in more uniform fiber [8,30]. However, if the rotation speeds are faster than the drawing speed of the fibers, fiber breakage may happen during the electrospinning process [30]. Thus, in this study, we set 2999 rpm as the rotation speed for a 9% (*w*/*v*) PCL solution. FESEM images revealed that 9% (*w*/*v*) PCL/CHCl_3_ emulsions were able to produce aligned nanofibers under controlled conditions.

#### 3.2.2. Analysis of Surface Hydrophilicity

**Figure 3:**
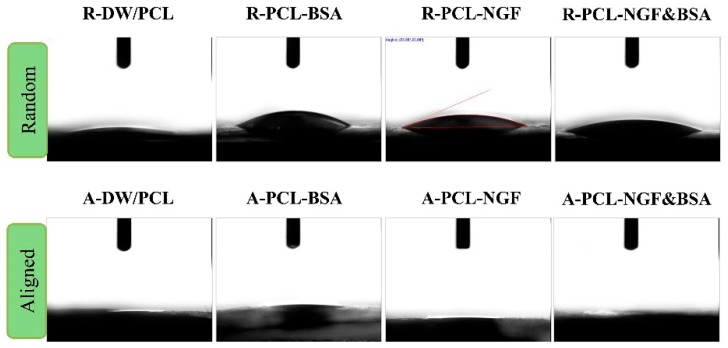
Water contact angle of emulsion electrospun PCL nanofibrous scaffolds after 12 s.

The surface hydrophilicity of the electrospun nanofibers plays an important role in determining nanofibrous mats properties as the drug delivery vehicles or tissue engineering scaffolds, and the water contact angle analysis has been regarded as a conventional method to test the hydrophilicity of nanofibrous mats [31]. Results of water contact angle measurement are summarized in Table 1 and Figure 3. From Table 1 and Figure 3, we can find that all the samples revealed extremely high hydrophilicity, which can be due to the addition of surfactant Span 80 that introduced hydrophilic group and significantly reduced the surface tension of the nanofibers. In addition, the hydrophilicity of emulsion electrospun aligned PCL nanofibers was higher than that of the random PCL nanofibers. As shown in Figure 3, the aligned nanofibrous mats were thoroughly wetted after 12 s contact so that its water contact angle was almost absent, while the water contact angle of random nanofibers were still visible (less than 30°), which can be attributed to the difference in the topography of nanofibers. When the water droplet touched the surface of aligned nanofibrous mats, the water droplet pervaded rapidly along the axial direction of the nanofibers and completely penetrated into fiber within 12 s (Figure 3). In terms of random nanofibers, the water droplet spread over every direction, which decreased the rate of droplet expansion. This result suggested that the hydrophilicity of nanofibrous mats was influenced by surface chemical properties and its topography.

#### 3.2.3. Study on the Internal Structure of Core-Shell Nanofibers

**Figure 4:**
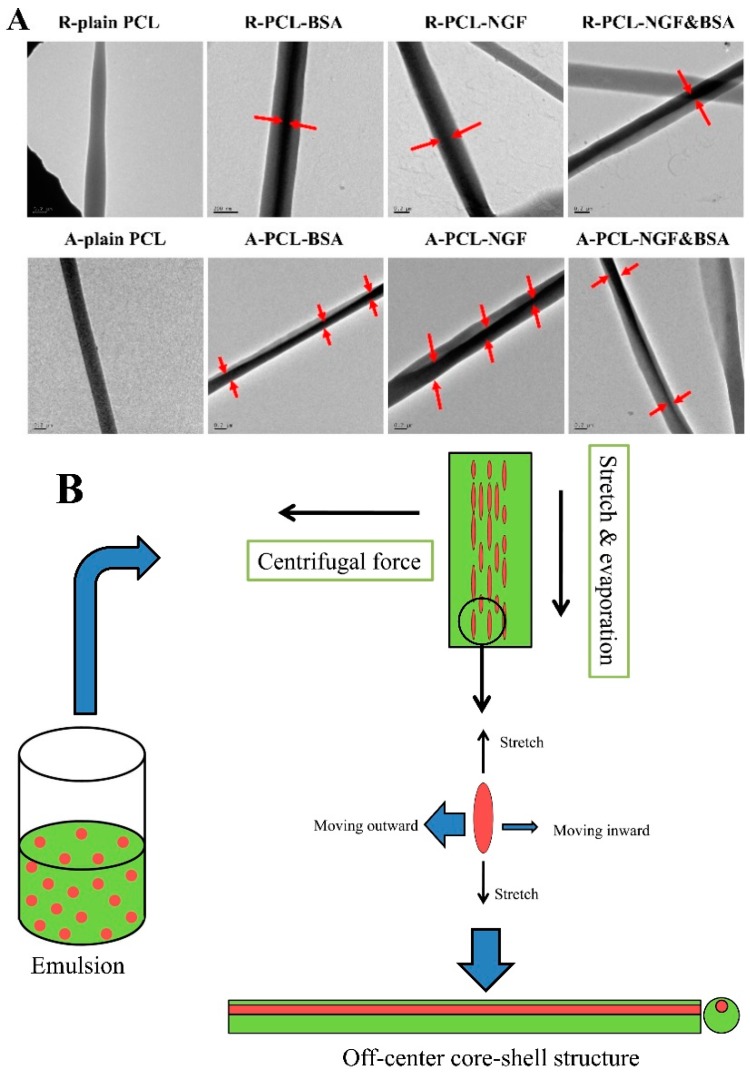
TEM images of PCL nanofibers and schematic diagram of “off-center” core-shell structure formation process. (**A**) TEM images of random and aligned plain PCL, PCL-BSA, PCL-NGF, and PCL-NGF&BSA nanofibers, scale bar = 0.2 μm; (**B**) Schematic illustration for the formation of “off-centre” core-shell fibers during emulsion electrospinning of aligned PCL nanofibers. Red color represents water phase and green color indicates oil phase.

Figure 4A reveals the core-shell structure of the protein encapsulated random and aligned PCL nanofibers prepared by emulsion electrospinning and the non-core-shell structure of the plain PCL nanofibers. It is obvious that the random nanofibers showed a relatively uniform core-shell structure with the protein (water phase) encased by PCL shell. While the core structure of the aligned nanofibers was not exactly in the center of nanofibers, revealing the “off-center” structure. We hypothesize that this phenomenon might be caused by the centrifugal force generated by the high-speed rotating drum. During the emulsion electrospinning process, the water phase was dispersed in oil phase, and chloroform evaporates relatively faster than distilled water, therefore, the viscosity of oil phase (the outer layer of the fiber) increases more rapidly than that of the water phase (inner layer of the fiber). It is the viscosity difference between oil phase and water phase, and the viscosity gradient from the outer layer to the inner layer that leads to the inward movement of the emulsion droplets and further results in the formation of core-shell structure [32,33]. This process is called “evaporation and stretching induced de-emulsification” [34]. In addition, the aligned emulsion electrospun nanofibers were prepared under an extremely unstable circumstance, where a high-voltage and a high speed rotating drum were applied to stretch the jet to thin nanofibers in a few seconds. We speculated that this unstable electrospinning process might lead to the variation of drug/protein (water phase) locations in the core-shell structure of aligned nanofibers. Actually, during the production of aligned fibers, many nanofibers were thrown onto the support platform of the rotating cylinder due to its centrifugal force, resulting in large waste of the encapsulated proteins. It is this centrifugal force of rotating drum that results in the movement of protein droplets from inner core to near surface of the fibers and leads to the formation of more “off-centre” structure and less concentric core-shell structure of protein loaded aligned nanofibers compared to that of random nanofibers collected on a static collector. This can be called as “centrifugal force induced inner core moving outward phenomenon”, and is described schematically in Figure 4B. The difference in the core-shell structures of the drug loaded random and aligned nanofibers might influence their drug release profiles.

### 3.3. Encapsulation Efficiency and In vitro Protein Release Analysis of BSA

The encapsulation efficiency of BSA in R-PCL-BSA and A-PCL-BSA nanofibers were 98.11% ± 4.63% and 78.84% ± 1.38%, respectively. The results show that the method for collecting fiber significantly influenced the encapsulation efficiency. During the preparation of aligned nanofibers, more proteins were lost, leading to the lower encapsulation efficiency of A-PCL-BSA scaffolds.

**Figure 5:**
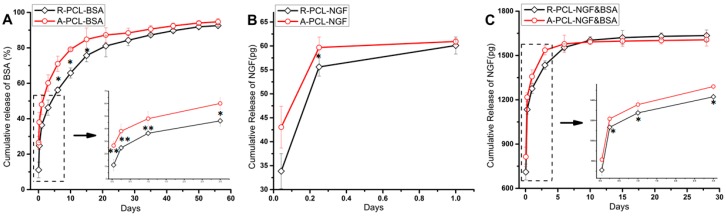
*In vitro* release profiles of BSA and NGF. (**A**) Release curve of BSA from (R/A)-PCL-BSA scaffolds; (**B**) Release properties of NGF from (R/A)-PCL-NGF scaffolds; (**C**) NGF released from (R/A)-PCL-NGF&BSA scaffolds. All of the data were expressed as means ± standard deviation, *n* = 3, ** *p* < 0.01, * *p* < 0.05.

It can be seen from Figure 5A that both the random and aligned nanofibers showed a sustained release of BSA lasted for 56 days. Obviously, random nanofibers (R-PCL-BSA) presented a slight initial burst release (11.09% ± 4.43%), which was about 15% lower than that of the A-PCL-BSA nanofibers (26.57% ± 3.52%) at the same time point (first 1 h). The initial burst release results from desorption of proteins loosely attached near or on the surface of the nanofibers. The higher initial burst release of A-PCL-BSA nanofibers compared to R-PCL-BSA nanofibers, indicated that more BSA escaped to the surface or near surface of A-PCL-BSA nanofibers rather than being encapsulated effectively into the core of them. The higher burst release of A-PCL-BSA nanofibers may be attributed the presence of “off-center” structures and the nanofibers with “off-center” structures had thin polymeric shell, as can be seen in Figure 4A. The thin polymeric barriers benefit water-soluble BSA to migrate to the aqueous medium. After initial burst release (first 72 h), BSA released from R-PCL-BSA nanofibers was followed by a sustained and constant release period of 56 days, in which the release percentage of BSA from A-PCL-BSA nanofibers were much higher than that of R-PCL-BSA in the first 30 days and became similar to the release percentage of R-PCL-BSA nanofibers after 42 days. Since PCL is quite hydrophobic and has a long degradation time (>2 years for complete degradation), the observed BSA release in this study was mainly due to diffusion of the protein out of the polymer matrix. The sustained release of BSA at the later stage is due to the increased diffusion path. During the period of 56 days of release, BSA released from R-PCL-BSA and A-PCL-BSA was 92.57% ± 0.41% and 94.72% ± 1.94%, respectively. The accumulative amount of BSA released from R-PCL-BSA and A-PCL-BSA nanofibers did not show much difference.

### 3.4. In vitro Release Study of NGF

Neurotrophic factor is essential in maintaining neural cells survival and promoting neurite outgrowth. An important topic in nerve tissue engineering is to repair the nerve defects after nerve injury. Keeping exogenous neurotrophic factors sustained release in nerve injury site is an important consideration during the design of tissue engineering scaffolds to efficiently combine neural tissue engineering and drug delivery systems. The combination of neural tissue engineering and drug delivery systems might mimic the *in vivo* release profiles of other kind of growth factors and neurotrophins during natural tissue self-repair [8]. Stimulating nerve growth at the injury site is the most important during the first 10~15 days of repair [12]. It takes two weeks for outgrowing axons to enter the conduit and approximately four weeks to traverse a 1 cm gap in the rat sciatic nerve injury [35]. Therefore, we focused on designing a core-shell structured NGF delivery system by emulsion electrospinning technique, which is capable of releasing bioactive NGF from PCL matrix for a period of four weeks.

The release of NGF from random and aligned emulsion electrospun PCL nanofibers was analyzed by NGF ELISA kit and is shown in Figure 5B,C. From Figure 5B we can find that the totally released amount of NGF from random nanofibers (R-PCL-NGF) and aligned nanofibers (A-PCL-NGF) were around 60 pg on the first day, which is much lower compared to the theoretically incorporation of NGF in the nanofibers. The initial release of NGF from the R-PCL-NGF nanofibers was slightly lower than that from the A-PCL-NGF nanofibers, and the cumulative release of NGF from R-PCL-NGF slowly approached that of the A-PCL-NGF nanofibers by the end of the first day. The amount of NGF released from both the R-PCL-NGF and A-PCL-NGF nanofibers cannot be detected after one day, indicating that some of the growth factor might be denatured or loss immunoreactivity during the fiber preparation procedure, as NGF has a limited stability under physiological conditions and its stability may be reduced in the case of protein encapsulation process by mechanical agitation [36].

Researchers have demonstrated the bioactivity of NGF released from PLGA nanospheres [8], BSA/PCL blend nanofibers [20], PLLACL nanofibers [23], PEGPCL hydrogel-PCL composites [37] and silk fibroin [7,38] over a 10 to 28 day period. The long stability of encapsulated NGF may result from a combination of several kinds of protein protective factors [38,39], including Tween 20 and BSA. Pfister, L.A. *et al.* [12] found that the addition of 0.05% Tween 20 in PBS (pH 7.4) with NGF improved both the responsiveness and consistency of the NGF assay. Although 0.05% Tween 20 was mixed with PBS in this study, the quantitative tests of NGF release from R-PCL-NGF and A-PCL-NGF scaffolds remain a challenge after one day. BSA is a globular protein with numerous biochemical applications and can be used as a stabilizer to prevent the denaturation of the growth factor during the nanofibers fabrication process [24]. Moreover, BSA is a commonly used release model protein for neural tissue engineering application [8,23]. Therefore, in this study, BSA was chosen as a protective agent, which can reduce the exposure of the NGF molecules to shear stress and organic solvent during the encapsulation process, accordingly prolong the bioactivity of NGF [24]. Figure 5C showed the release profiles of NGF from the R-PCL-NGF&BSA and A-PCL-NGF&BSA scaffolds. With the addition of stabilizer BSA to water phase, the NGF release from random and aligned NGF&BSA nanofibers was extended over 28 days. A relatively higher initial burst release of NGF from A-PCL-NGF&BSA nanofibers was shown in Figure 5C, which is 813.73 ± 0.32 pg, while the NGF released from R-PCL-NGF&BSA nanofibers was 710.87 ± 0.27 pg in the first hour. After burst release of first hour, the NGF released from (R/A)-PCL-NGF&BSA nanofibers was followed by a sustained and constant release of 28 days period. In the first 10 days, the cumulative release of NGF from R-PCL-NGF&BSA nanofibers was relatively lower than that of A-PCL-NGF&BSA nanofibers, while after 10 days, the released amount of NGF from R-PCL-NGF&BSA slightly exceeded A-PCL-NGF&BSA nanofibers. Compared to R-PCL-NGF&BSA nanofibers, the higher burst release of A-PCL-NGF&BSA nanofibers might be due to presence of “off-center” core-shell structures in aligned fibers. While the slightly higher release amount of NGF from R-PCL-NGF&BSA scaffold after 10 days might be attributed to the higher encapsulation efficiency of NGF in R-PCL-NGF&BSA scaffold than that of A-PCL-NGF&BSA nanofibers, which can be confirmed by the former encapsulation efficiency study of BSA in (R/A)-PCL-BSA nanofibers. After 15 days, the release of NGF continued until the end of the 28 days with a relative low daily release from the (R/A)-PCL-NGF&BSA nanofibrous mats. The NGF content in release medium collected after 28 days was too low to be detected using an ELISA kit. Some scholars believe that the limited duration of release testing can be due to the instability of NGF over extended incubation times [38]. We consider that the limited duration of NGF release in this study may be related to low loading of NGF. From the preceding discussion, the first 10~15 days after nerve injury is the most important for the nerve repair [12]. In this study, the release results of NGF from (R/A)-PCL-NGF&BSA nanofibrous scaffolds can satisfy the requirement in the nerve regeneration.

### 3.5. In vitro Cytotoxicity Study of Emulsion Electrospun Nanofibers

**Figure 6:**
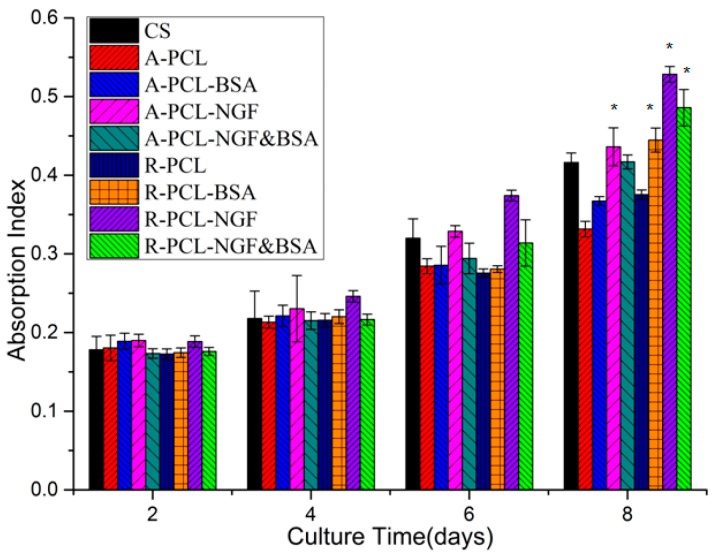
MTS assay of PC12 cells viability on CS and emulsion electrospun PCL nanofibrous scaffold (* = significantly different in comparison with CS, *p* < 0.05, *n* = 3).

The MTS assay is an important method for evaluating the *in vitro* cytotoxicity of biomaterials. From a clinical point of view, an ideal drug incorporated tissue engineering scaffold must have a good biocompatibility with tissue cells and a sustained controlled drug release. Figure 6 depicts the MTS assay profiles of PC12 cells viability on CS, (R/A)-PCL, (R/A)-PCL-BSA, (R/A)-PCL-NGF, and (R/A)-PCL-NGF&BSA. The MTS assay showed that the number of live cell on all scaffolds and CS increased with the prolonging of incubation time, and among all the eight scaffolds, the R-PCL-NGF and R-PCL-NGF&BSA nanofibers have the most number of live cells and the A-PCL nanofibers showed the least live cell numbers. Also, the MTS assay exhibited that no significant difference in the number of cells on day 2; by day 8, the number of cell viability on (R/A)-PCL-NGF, R-PCL-NGF&BSA and R-PCL-BSA surpassed that on CS. The results showed that the incorporation of NGF or NGF&BSA did not show any adverse effects on PC 12 cells survival, suggesting that the (R/A)-PCL-NGF and (R/A)-PCL-NGF&BSA scaffolds have the potential for neural tissue engineering application.

### 3.6. Differentiation Study of PC12 Cells

#### 3.6.1. Morphology Analysis of Differentiated PC12 Cells

**Figure 7:**
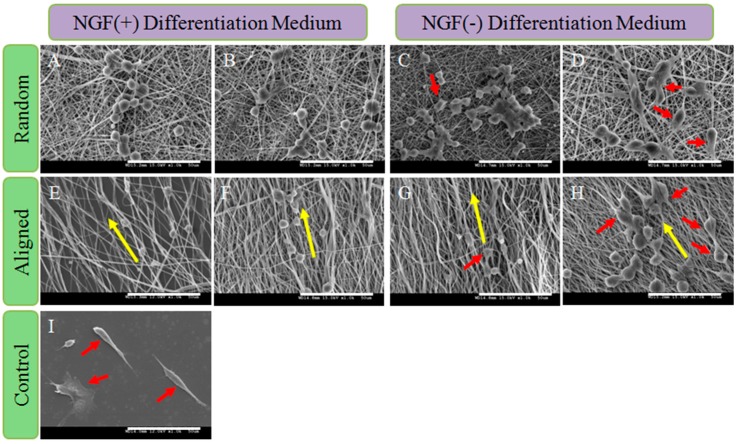
SEM images of the differentiated PC12 cells on (**A**) R-PCL; (**B**) R-PCL-BSA; (**C**) R-PCL-NGF; (**D**) R-PCL-NGF&BSA; (**E**) A-PCL; (**F**) A-PCL-BSA; (**G**) A-PCL-NGF; (**H**) A-PCL-NGF&BSA and (**I**) CS after stimulated for eight days; scale bar = 10 mm. Notes: Yellow arrow indicates the orientation of aligned fiber and red arrow shows the neurite-bearing PC12 cells.

PC12 cells were used to evaluate the synergistic effect of topographic cue and nerve growth factor (NGF) on neuron growth. Figure 7 displayed the SEM images of the differentiated PC12 cells grown on nanofibrous scaffolds. The cells were allowed to adhere to the substrate for 24 h before being stimulated with NGF. The undifferentiated PC12 cells were small and spherical. After eight days of culture, a few PC12 cells grown on NGF-added (R/A)-PCL, (R/A)-PCL-BSA scaffolds showed an elongated shape, while on (R/A)-PCL-NGF and (R/A)-PCL-NGF&BSA scaffolds, there were more PC12 cells projected the neurites. These results suggested that NGF could stimulate PC12 cell differentiation but the stimulation of NGF released from the NGF encapsulated nanofibers were more effective on PC12 cell differentiation than that of NGF directly added in culture medium.

#### 3.6.2. Analysis of the Expression of α-Tubulin

**Figure 8:**
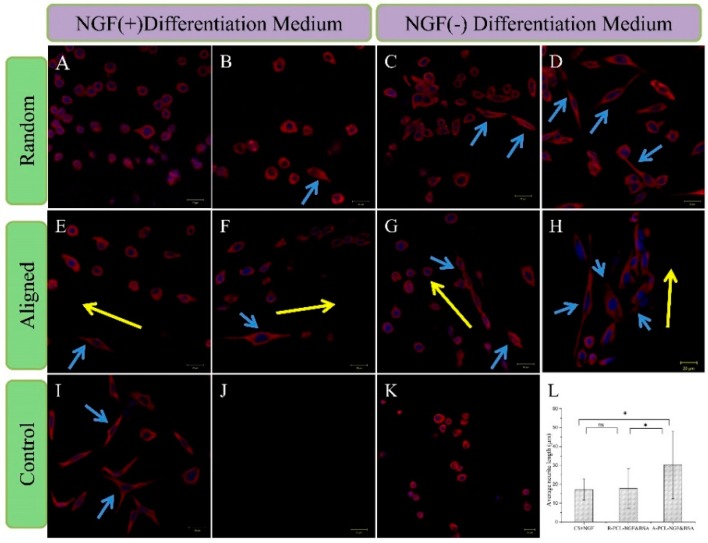
Fluorescent images of PC12 cells cultured for eight days on the surface of different samples with labeling of cytoplasm (red) and nuclei (blue). (**A**) R-PCL; (**B**) R-PCL-BSA; (**C**) R-PCL-NGF; (**D**) R-PCL-NGF&BSA; (**E**) A-PCL; (**F**) A-PCL-BSA; (**G**) A-PCL-NGF; (**H**) A-PCL-NGF&BSA; (**I**) CS-positive; (**J**) CS-blank for immunofluorescence staining; (**K**) CS-negative; scale bar = 20 μm. (**L**) Neurite length of PC12 cells on CS-positive, R-PCL-NGF&BSA and A-PCL-NGF&BSA after eight days cell culture. Neurite length was measured using ImageJ software. Data were mean ± standard deviation; *n* = 10; ns: no significance; * *p* < 0.05. Notes: Yellow arrow indicates the alignment direction for the underlying nanofibers and blue arrow shows the neurite-bearing PC12 cells.

α-Tubulin is a kind of cytoskeletal protein in neurons and plays an essential role in extension and maintenance of axonal and dendritic processes. After eight days of culture, the expression of the α-Tubulin in PC12 cells was analyzed by immunofluorescence staining to further evaluate effects of NGF on PC12 cell differentiation. From Figure 8, we can find out that the cells cultured on different scaffolds all expressed α-Tubulin protein. Figure 8(K) is the LSCM image of PC12 cells cultured on CS without the addition of NGF in culture medium. Under the stimulated condition by directly adding NGF to the culture medium, most PC12 cells on CS showed a neuron-like appearance with bigger soma and well grown neurites (Figure 8I). Meanwhile, PC12 cells grown on (R/A)-PCL-NGF and (R/A)-PCL-NGF&BSA scaffolds showed more neurites formed compared to that on the (R/A)-PCL and (R/A)-PCL-BSA scaffolds. These results indicated that NGF released from (R/A)-PCL-NGF and (R/A)-PCL-NGF&BSA, especially from the (R/A)-PCL-NGF&BSA scaffolds was sufficient and active enough to support the neuronal differentiation.

To evaluate the differentiation of PC12 cells quantitatively, the neurite length of differentiated PC12 cells were calculated. The neurite length is defined as the linear distance from the tip of the neurite to the cell junction. The average neurite length was used to compare the differentiation level of PC12 cells grown on CS-positive control, R-PCL-NGF&BSA and A-PCL-NGF&BSA scaffolds. Under the stimulated condition by directly adding NGF, the average neurite length of the PC12 cells on CS was 17.22 ± 5.56 μm and their longest neurite was up to 27.32 μm (Figure 8I,L). The mean length of neurite projecting on R-PCL-NGF&BSA nanofibers was 17.86 ± 10.39 μm, with an increase in maximum length of neurite being observed: 41.67 μm (Figure 8D,L). The mean neurite length of PC12 cells on A-PCL-NGF&BSA scaffold was the maximum (30.33 ± 17.92 μm) among all samples, with a significantly enhanced maximum neurite length (70.17 μm) compared to that on R-PCL-NGF&BSA nanofibers (41.67 μm) and CS (Figure 8H,L). This may be attributed to the different hydrophilicity between A-PCL-NGF&BSA scaffold and R-PCL-NGF&BSA nanofibers (Figure 3). As the hydrophilic property of the biomaterial is the key factor that influences the initial behavior of the material with cells. Materials with good hydrophilicity can promote cell adhesion, which further determines the survival, proliferation, and differentiation of the cells. Current research shows that aligned nanofibers can induce nerve cells along the fiber axis migration and enhance the cell adhesion [40], which has special significance for nerve repair because of the orientation growth of nerve cells being able to accelerate the functional nerve regeneration.

Some scholars believe that the sustained release of a small amount of NGF from the culture matrix could bind to receptors on the cultured cells much more efficiently than NGF directly added to the culture medium [41]. A similar trend was also found in this study. PC12 cells cultured on (R/A)-PCL-NGF&BSA with the released NGF at low nanogram range showed better differentiation compared to that on CS-positive control which was cultured under stimulated condition with the addition of 50 ng of NGF every two days in 1 mL Differentiation Medium (totally 200 ng of NGF treatment over eight days). Actually, the nanfibers collected on coverslips were very thin. We assumed that the weights of the fibers on the coverslips for cell culture were the same as that of release study (10 mg), from which about 1600 pg of NGF released out into the PC12 cells Differentiation Medium over eight days (Figure 5C). The result suggested that the sustained release of a small amount of NGF from (R/A)-PCL-NGF&BSA scaffolds was enough to induce the neuronal differentiation and NGF&BSA incorporated random and aligned core-shell nanofibrous scaffolds prepared by emulsion electrospinning has a potential for guiding nerve tissue growth and promoting nerve regeneration.

## 4. Conclusions

The enhancement of axon regeneration ability, guidance for the extension of the axon and cell migration must be taken into account in the design of tissue engineering scaffold for neural repair. In this study, we prepared random and aligned NGF loaded PCL nanofibrous scaffold via emulsion electrospinning technique, and investigated their feasibility as a drug delivery system and nerve tissue engineering scaffold. The release of BSA from (R/A)-PCL-BSA sustained for 56 days. The sustained release of NGF from (R/A)-PCL-NGF&BSA was detected for 28 days. All the scaffolds showed good biocompatibility and had no side effects on the survival of PC12 cells, while more cell viability on (R/A)-PCL-NGF and (R/A)-PCL-NGF&BSA scaffolds was observed compared to other scaffolds. The sustained release of a small amount of NGF from (R/A)-PCL-NGF&BSA scaffolds was enough to induce the neuronal differentiation. The topographical cue of aligned nanofibrous scaffold had a positive effect on PC12 cells differentiation in which neurite extension was axially aligned and PC12 cells on A-PCL-NGF&BSA nanofibers possessed the longest neurite of 70.17 μm by day 8. NGF release and fiber orientation could exert a synergistic effect on PC12 cell differentiation. Our studies demonstrated that the (R/A)-PCL-NGF&BSA core-shell nanofibrous scaffolds could create a cell-favorable microenvironment, which not only can mimic the natural ECM but also serve as a model drug delivery system for the sustained release of NGF. All these results could contribute to a better design of a nerve tissue scaffold with incorporation of the synergistic effect of topographic and feasible biochemical cues for nerve injury repair.

## Figures and Tables

**Table 1:** Diameter and water contact angle of emulsion electrospun nanofibers.

Scaffolds (8% PCL)	W/O	Diameter (nm/n = 100)	Contact angle (°/n = 3)	Scaffolds (9% PCL)	W/O	Diameter (nm/n = 100)	Contact angle (°/n = 3)
R-PCL	DW-PCL	343 ± 113	0	A-PCL	DW-PCL	354 ± 91	0
R-PCL-BSA	BSA-PCL	301 ± 76	24.7 ± 1.2	A-PCL-BSA	BSA-PCL	302 ± 70	6.7 ± 2.5
R-PCL-NGF	NGF-PCL	371 ± 95	22.8 ± 2.9	A-PCL-NGF	NGF-PCL	333 ± 90	0
R-PCL-NGF&BSA	NGF&BSA-PCL	320 ± 97	21.4 ± 1.7	A-PCL-NGF&BSA	NGF&BSA-PCL	320 ± 87	0

Note: DW is the abbreviation of distilled water.
